# Enhancement of growth and salt tolerance in *Clematis chinensis* by the plant growth- promoting fungus *Trichoderma compactum* SZ01

**DOI:** 10.1186/s12870-026-09648-7

**Published:** 2026-07-31

**Authors:** Yucui Wu, Xuebo Xue, Shichao Zhang, Yuxuan Rong, Yaqian Sun, Wenqi Liu, Libo Yang, Menghua Wang, Tao Jiang

**Affiliations:** 1 School of Landscape and Ecological Engineering, Hebei University of Engineering, Handan, 056038 China; 2 Institute of Cash Crops, Hebei Academy of Agricultural and Forestry Sciences, Shijiazhuang, 050051 China; 3 Department of Natural Medicinal Chemistry, School of Traditional Chinese Pharmacy, China Pharmaceutical University, Nanjing, 211198 China; 4 National Key Laboratory of Crop Genetic Improvement, Huazhong Agricultural University, Wuhan, 430070 China

**Keywords:** *Clematis chinensis*, *Trichoderma compactum*, Salt tolerance, Transcriptome, Plant-microbe interaction

## Abstract

**Background:**

Salt-tolerant rhizosphere microorganisms with multiple plant growth–promoting (PGP) traits represent cost-effective biological inoculants for enhancing plant tolerance to abiotic stress.

**Results:**

In this study, a salt-tolerant fungal strain, SZ01, was isolated from the rhizosphere of *Clematis chinensis* using PDA medium supplemented with 1% NaCl and identified as *Trichoderma compactum* based on morphological and molecular analyses. SZ01 tolerated NaCl concentrations up to 9%. Functional characterization revealed that SZ01 exhibited potassium solubilization, nitrogen fixation potential, and siderophore production, although it lacked the ability to solubilize organic or inorganic phosphorus. Pot experiments demonstrated that under salt stress, inoculation with SZ01 spore suspension significantly increased the fresh and dry weights of underground tissues, reduced Malondialdehyde (MDA) accumulation, and enhanced soluble sugar and proline contents compared with non-inoculated controls, indicating effective mitigation of salt-induced physiological damage. Transcriptomic analysis of *C. chinensis* leaves further showed that SZ01 treatment triggered extensive transcriptional reprogramming, particularly through modulation of MAPK and metabolic pathways, thereby optimizing stress-responsive gene expression. Secondary metabolite profiling of SZ01 under salt stress revealed the accumulation of chlorine-containing molecules potentially contributing to plant growth promotion.

**Conclusion:**

These findings highlight the potential of SZ01 as a microbial inoculant to promote plant growth and productivity in saline–alkaline environments, with implications for both medicinal and agricultural crop production.

**Supplementary Information:**

The online version contains supplementary material available at https://doi.org/10.1186/s12870-026-09648-7.

## Introduction

 Soil salinization is one of the most severe global environmental issues currently. In recent years, climate change, seawater backflow, groundwater infiltration, and human-being activities such as irrigation and fertilizer application increased salt concentration in soil, resulting in soil salinization [1]. Salinization is estimated to affect one-fourth to one-third crop production worldwide [2]. Salt stress adversely impacts plant growth and development by hindering seed germination, limiting water and nutrients uptake [3, 4]. This subsequently triggers osmotic stress and ionic toxicity, resulting in physiological drought, metabolic disorders, and suppressed photosynthesis in plants. High-salt stress ultimately leads to stunted growth, leaf wilting, reduced both yield and quality, and even plant death. Salinity stress also affects soil microbiota adversely as well as the physical and chemical properties of soil, thereby critically compromising overall soil health [5]. However, plants must refigure their physiological and biochemical processes and adjust their developmental and metabolic paradigm to adapt to saline conditions [4]. For example, plants overcome salinity stress by restoring osmotic and ionic homeostasis, improving the synthesis of phytohormones and osmoprotectants, and upregulating antioxidant activities [6].

Currently, another strategy of enhancing plant productivity and stress resilience is to cohabit with diverse halo tolerant microorganisms, such as rhizosphere, epiphytes and endophytes [7, 8]. Halo tolerant microorganisms enhance plant resilience to salt stress through diverse mechanisms by modulating phytohormones, enhancing ion homoeostasis, improving nutrient uptake, and promoting root growth [9–11]. Several studies have found that inoculation with halotolerant microorganisms was vindicated and highly effective in inducing salinity tolerance in plants [12]. Jin et al. found that halotolerant microorganism-based soil conditioner application improved the yield, the quality, and starch characteristics of rice under salt stress conditions [13]. Saha et al. reported that *Trichoderma harzianum* treated plant showed a lower accumulation of H_2_O_2_ and MDA, but a higher antioxidant enzymes and chlorophyll content were found under saline conditions [14].

Therefore, the development and utilization of rhizosphere salt-tolerant plant growth-promoting microorganisms represent a safe, efficient, and environmentally friendly approach for saline soil remediation, holding broad prospects for application.

*Clematis chinensis* Osbeck is a widely used traditional Chinese medicinal plant to treat various diseases/disorders, such as relieving rheumatism pain and scapulohumeral periarthritis [15]. In recent years, researchers have focused on the pharmacological effects of *C. chinensis*, particularly its antimicrobial, anti-inflammatory, analgesic, and antitumor activities [16]. Our previous investigation found that *C. chinensis* exhibits a degree of salt tolerance and can grow in saline–alkali soils. In this context, our aim was to isolate newly native salt-tolerant fungi from the rhizosphere of *C. chinensis* growing in saline-alkali soil and to assess their ability to produce growth-promoting substances under salinity stress. The study provides both a microbial resource and a theoretical basis for the remediation of salinized soils, thereby advancing the cultivation of Chinese medicinal plants in saline–alkali soil.

## Materials and methods

### Sample collection and fungi isolation

The soil surrounding the roots of *C. chinensis* Osbeck growing in coastal saline-alkali soil were collected from the state-owned farm in Haixing County, Cangzhou City, Hebei Province, China (38°21′N, 117°31′E). The soil samples were processed using serial dilution and spread plate method [17]. For emergence of fungi, the diluted sample suspensions were placed on Petri dishes with potato dextrose agar (PDA) containing 1% NaCl, allowed to dry well, and then cultured for 5 days at 28 °C. Following repeated subculturing of mycelial isolates on PDA plates, a pure culture was obtained by the hyphal tip method and designated SZ01.

### Morphological and molecular identification

The isolated SZ01 fungus was inoculated onto PDA plates and incubated in the dark at 28 °C for 5 days. The colony color, shape, and morphology were noted. Additionally, scanning electron microscopy (SEM) observations were conducted to examine the characteristics such as the shapes of hyphae and spores for preliminary identification.

The isolated SZ01 fungus was identified through internal transcribed spacer (*ITS*) rRNA gene and RNA polymerase subunit II (*RPB2*) gene analysis. PCR reactions were performed with two primer sets, ITS1 (5’-TCCGTAGGTGAACCTGCGG-3’) and ITS4 (5’-TCCTCCGCTTATTGATATGC-3’), and RPB2-F (GAYGAYMGWGATCAYTTYGG) and RPB2-R(CCCATRGCTTGYTTRCCCAT) [18]. The sequences obtained were analyzed using the BLAST tool available at the National Center for Biotechnology Information. The multiple sequence alignment of the target sequences was performed using the MAFFT software with the default parameters. Subsequently, phylogenetic relationships were determined using the Maximum-likelihood (ML) method in the MEGA11 software [19], with 1000 bootstrap replications to evaluate branch reliability.

### Evaluating the effects of salinity on growth of SZ01

To assess the effect of salinity on SZ01 growth, the mycelium was inoculated on PDA medium supplemented with NaCl concentrations of 5%, 6%, 7%, 8%, 9%, and 10%, respectively. Each treatment was conducted with three replicates, and PDA plates without NaCl served as the control. The isolated fungus was cultured in petri dishes and incubated at 28 °C. The fungal growth was monitored every 3 days for 15 days. The fungal growth was rated on a four-point scale: excellent growth with no discernible difference from the control (+++); growth that was slightly reduced, showing diminished sporulation, altered aerial-mycelium colour, or sparser density (++); growth devoid of sporulation or aerial mycelium (+); and complete absence of growth (−).

### Plant growth-promoting characteristics of SZ01

Phosphate solubilization by the isolate was carried out on organic phosphorus and inorganic phosphorus media with three replicates, respectively. The formation of halo zone was examined to assess phosphate -solubilizing ability of SZ01 [20]. We used the classic CAS method to detect siderophores of SZ01 and then examine whether an orange-yellow halo appears on the medium [21]. The nitrogen-fixing ability of SZ01 was assessed by culturing the strain on Ashby’s medium and then observing its growth [22]. All the plates were incubated for 5 days at 28 °C. Potassium solubilization by SZ01 was tested on PDA medium supplemented with a small amount of feldspar as the sole K source. The plates were then incubated for 3 days at 28 °C. We then recorded the chemotactic response of the mycelium on the plate whether toward feldspar to assess the potassium-solubilizing activity of SZ01 [23].

### Preparation of plant material and execution of growth assays

One-year-old seedlings of *C. chinensis* were obtained from the germplasm resource center of the Institute of Cash Crops, Hebei Academy of Agricultural and Forestry Sciences (HAAFS), China. The plant materials were formally identified by Dr. Tao Jiang (HAAFS). Voucher specimens were deposited in the Herbarium of HAAFS (accession number: WLX-2024-009). The seedlings were transplanted into nutrient pots (height: 25 cm; diameter: 20 cm) and cultivated for 20 days under consistent greenhouse conditions. Seedlings of comparable size were selected for the experiment. All experiments complied with institutional and national guidelines for plant research. The experiment was conducted from June to August 2024 in the greenhouse of HAAFS (114.45° E, 38.06° N). Greenhouse conditions: 25–28 °C; 60%-75% relative humidity; natural photoperiod.

The conidia suspensions SZ01 were prepared according to the method of Zhang et al. [24]. In the preliminary experiment, a salt treatment group was simultaneously treated with 10 mL of spore suspensions at 0, 10⁵, 10⁶, 10⁷, 10⁸, and 10⁹ conidia·mL⁻¹ every 7 days and 200 mL of 0.60% NaCl solution every 5 days. Under 0.6% salt stress, the 10⁸ conidia·mL⁻¹ spore suspension most significantly improved the physiological indices of *C. chinensis* and was thus selected for subsequent experiments(Fig. S1).

The formal experimental setups were divided into a control group and a spore-treated group. The control group: sterile water treated seedlings with NaCl (0, 0.40%, 0.60%, and 0.80%). The spore-treated group: SZ01-treated *C. chinensis* seedlings with NaCl (0, 0.40%, 0.60%, and 0.80%). *C. chinensis* Seedlings were irrigated with 200 mL NaCl solution every 5 days. 10 mL of SZ01 conidia suspensions (10⁸ conidia·mL⁻¹) were applied every 7 days, while the water control group received in 10 mL of sterile water instead. Three independent biological replicates were assigned to each treatment following a completely randomized design. The morphological, physiological and biochemical parameters of *C. chinensis* seedlings were investigated at 30 days after treatment. Roots of *C. chinensis* seedlings were removed, washed three times with distilled water, dried, and weighed. Root weights were measured using weighing balance.

### Physiological and biochemical characteristics in *C. chinensis* seedlings

The leaves of the same parts of *C. chinensis* seedlings were used to determine various physiological and biochemical parameters. Each treatment and control were.

repeated three times.

The MDA content in samples were investigated by the thiobarbituric acid (TBA) reaction as described in the method of Heath & Packer [25]. Leaf tissue (0.1 g) was weighed and homogenized with 1 mL of 5% (w/v) trichloroacetic acid (TCA) solution. The mixture was centrifuged at 10,000 rpm for 15 min. The supernatant was collected and mixed with an equal volume (1:1) of 0.67% (w/v) TBA solution, and then incubated at 95 °C in a water bath for 30 min. The absorbance of the reaction mixture was measured at 450 nm, 532 nm, and 600 nm using spectrophotometer.

The soluble sugar (SS) content in samples were investigated following the method of Yemm & Willis [26]. Leaf tissue (0.1 g) was weighed and homogenized with 1 mL of deionized water, followed by heating at 95 °C in a water bath for 10 min. After cooling to room temperature, the mixture was centrifuged at 10,000 rpm for 15 min. 40 µL of the supernatant was mixed with 40 µL deionized water, 20 µL anthrone-ethyl acetate reagent, and 200 µL concentrated sulfuric acid. The reaction mixture was then incubated at 95 °C for 10 min, cooled to room temperature, and absorbance was measured at 620 nm.

The proline (Pro) content and the antioxidant activities of superoxide dismutase (SOD) and peroxidase (POD) were determined according to the protocols provided in the Pro Content Assay Kit, SOD Activity Assay Kit, and POD Activity Assay Kit, respectively (Solarbio, Beijing, China). Pro was measured at 520 nm, SOD at 560 nm, and POD at 470 nm, respectively using spectrophotometer.

### RNA-Seq

We selected two time points, 10 days and 30 days, to collect leaves of *C. chinensis* subjected to four treatments: 0.6% NaCl treatment (NA), water treatment (CK), 0.6% NaCl + 10⁸ conidia·mL⁻¹ SZ01 spore treatment (NB), and water + 10⁸ conidia·mL⁻¹ SZ01 spore treatment (BZ). Each treatment group included three biological replicates. The collected samples were immediately frozen in liquid nitrogen and subsequently sent to Biomarker Technologies Corporation for RNA extraction and transcriptome sequencing.

For the subsequent differential expression gene (DEG) analysis, the comparative pairs were named using the “A vs B” format, where A represents the control group and B represents the experimental treatment group. According to the relative expression levels between the two groups of samples, DEGs were classified into up-regulated genes and down-regulated genes: up-regulated genes refer to those with higher expression levels in group B than in group A, while down-regulated genes are the opposite. Notably, the classification of up-regulation and down-regulation is relative, determined by the specified order of A (control) and B (treatment) in each comparison.

### Library construction and sequencing for RNA-seq

RNA was extracted using the Biomarker Plant Total RNA Isolation Kit (Biomarker Technologies Corporation, Beijing, China). All RNA samples underwent rigorous quality assessment. RNA purity and concentration were measured using a NanoDrop 2000 spectrophotometer, and RNA integrity was verified with an Agilent 2100 Bioanalyzer system (RIN > 7.0). Only samples passing quality control were used in subsequent experiments.

Eukaryotic mRNA was enriched using oligo (dT) magnetic beads and subsequently fragmented by adding Fragmentation Buffer. Using this mRNA as template, first-strand and second-strand cDNA were synthesized and purified. The purified double-stranded cDNA underwent end repair, polyadenylation, and ligation to sequencing adapters, followed by size selection using AMPure XP beads. Final cDNA libraries were obtained through PCR amplification.

Library quality was controlled through multiple steps: initial quantification was performed using Qubit 3.0, insert size was confirmed with the Qsep400 analysis system, and effective library concentration was precisely quantified (> 2 nM) using qPCR to ensure library quality. All libraries were sequenced on the Illumina high-throughput sequencing platform with PE150 configuration.

### RNA-seq data analysis

Following sequencing, raw reads were subjected to stringent quality control and preprocessing using Trimmomatic (v0.39) [27]. This process involved removing adapter-containing reads and eliminating low-quality sequences, specifically those with over 10% undefined bases (N) or where more than 50% of the bases possessed a quality score (Q) of 10 or lower. After this rigorous filtering, a total of 163.86 Gb of high-quality clean data was obtained, with the Q30 base percentage reaching no less than 97.64% across all samples. De novo assembly of these clean reads from all samples was performed with Trinity, yielding 98,952 unigenes with an N50 of 1,462 bp, indicating a high level of assembly completeness [28]. For functional annotation, unigene sequences were aligned against multiple databases (NR, Swiss-Prot, COG, KOG, eggNOG, KEGG) using DIAMOND [29]. KEGG orthology assignments were generated with KOBAS, while GO orthology was determined using InterProScan with the InterPro database. Following amino acid sequence prediction, domain searches were conducted with HMMER against the Pfam database [30, 31]. Applying stringent cutoffs (E-value ≤ 1e-5 for BLAST-based searches and ≤ 1e-10 for HMMER), 38,875 unigenes were successfully annotated. Finally, expression levels were quantified by aligning the clean reads back to the unigene library using Bowtie and estimating abundance as FPKM (Fragments Per Kilobase per Million mapped reads) with RSEM [32, 33].

### Principal component analysis

To evaluate sample reproducibility, we performed dimensionality reduction through Principal Component Analysis (PCA) on the count expression matrix of unigenes common to all samples [34]. Using the normalized FPKM expression matrix of all unigenes from 24 *C. chinensis* samples as input, we first corrected the gene FPKM expression values to eliminate biases caused by gene length and sequencing depth. PCA was then performed using the vegan package in R, and a scatter plot was generated with the ggplot2 package to visualize the similarity of transcriptome profiles among samples. Each dot represents one sample, with the same treatment group shown in the same color. The percentage values on the axes indicate the proportion of overall transcriptomic variance explained by each principal component. This analysis identified the primary patterns of sample distribution within the complex dataset, allowing for a clear assessment of the dispersion and grouping of biological replicates, thereby effectively demonstrating the high consistency between replicate samples.

### Differential expression analysis

Differential expression analysis was performed to identify significantly dysregulated genes between compared groups. Gene count values across all samples served as the input for differential analysis software, with the method selection based on experimental design. For comparisons involving groups with biological replicates, the DESeq2 package was employed, while the EBSeq algorithm was used for comparisons lacking biological replicates [35, 36]. Significantly DEGs were identified using a stringent threshold of fold change ≥ 2 (absolute log_2_FoldChange ≥ 1) and a False Discovery Rate (FDR) < 0.01. The fold change represents the ratio of expression levels between two samples or groups, while the FDR, obtained by adjusting the raw p-value for multiple testing, indicates the statistical significance of the observed differences. For comparative purposes, fold change values were logarithmically transformed to log_2_FC, where genes with larger absolute log_2_FC values and smaller FDR values demonstrate more pronounced differential expression between the sample groups.

### qRT-PCR validation of transcriptome

To verify the reliability of gene expression trends obtained from RNA-seq data, representative samples including CK10, BZ10, NA30 and NB30 were selected for qRT-PCR analysis of five differentially expressed genes and the reference gene Actin. All primer sequences are listed in Table S1. Total RNA retained from transcriptome sequencing was reverse-transcribed using the PerfectStart Uni RT&qPCR Kit. The qRT-PCR amplification program was set as follows: initial denaturation at 94 °C for 30 s, followed by 40 cycles of 94 °C for 5 s and 60 °C for 30 s. Three biological replicates were prepared for each sample, and the relative expression levels of target genes were calculated using the 2^−ΔΔCt^ method with CK10 as the calibrator. The gene expression trends determined by qRT-PCR were consistent with the FPKM values from RNA-seq, confirming the reliability of the transcriptome data (Fig. S4).

### LC-MS profiling of secondary metabolites produced by SZ01 under salt stress

The normal cultural medium of strain SZ01 was prepared by autoclaving rice (60 g) and water (80 mL) in a 500 mL flask. For salt stress conditions, the normal medium was supplemented with 5% NaCl. SZ01 was inoculated into sterile media for both control and salt stress groups and cultured for 30 days at room temperature. The resulting cultures were extracted three times with an equal volume of ethyl acetate. The combined organic extracts were concentrated under reduced pressure and redissolved in methanol. Prior to LC-MS analysis, the samples were centrifuged at 13,000 rpm for 5 min and filtered through a 0.22 μm membrane. The LC-HRMS system used for product analysis was a Thermo Orbitrap Exploris 120, equipped with a Hyperil Gold AQ column (100 mm×2.1 mm, 1.9 μm). The mobile phases consisted of: (A) water with 0.1% formic acid and (B) acetonitrile with 0.1% formic acid. The elution program was as follows: initial 20%B for 5 min, followed by a linear gradient to 95% B over 15 min, maintained at 95% for 5 min, and then returned to 20% B, with a constant flow rate of 0.5 mL/min. Data analysis was performed with Compound Discoverer.

### Data Analysis

Data were organized in tables using Microsoft Word 2019, and graphs were generated with GraphPad Prism 8.2.1. Values in the figures are presented as mean ± standard error. Correlation analysis and one-way ANOVA were performed using SPSS 20.0 (SPSS Inc., Chicago, IL, USA).

## Results

### Isolation and identification of the fungal strain

**Fig. 1:**
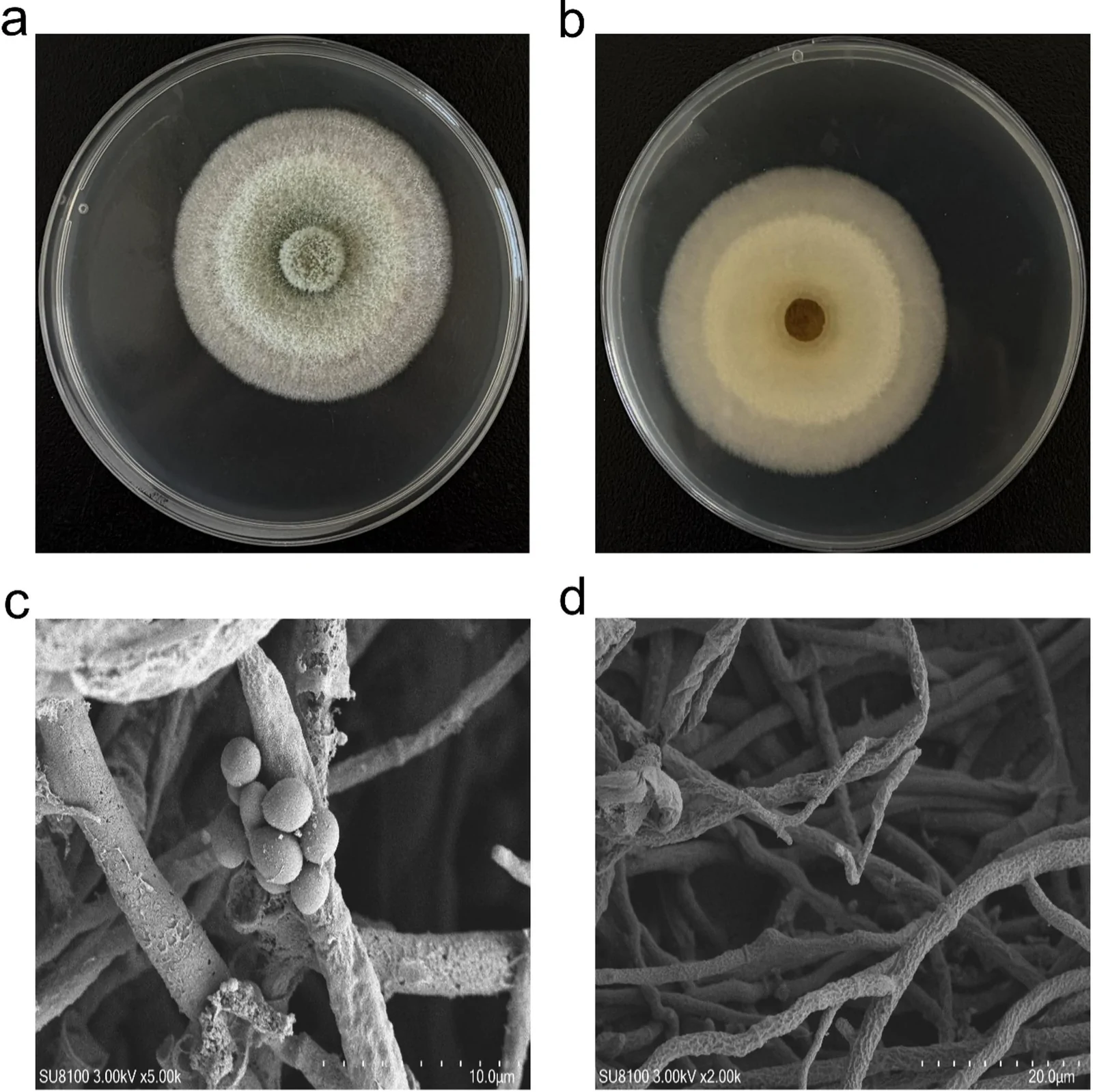
Morphological characteristics and ultrastructure of SZ01. **a** Front colony morphology; (**b**) Reverse colony morphology; (**c**) Spore ultrastructure (SEM); (**d**) Hyphal ultrastructure (SEM)

After the preliminary screening of soil suspensions on salt-containing PDA media, successive isolation and purification yielded a distinct fungal strain, designated as SZ01. When cultured on PDA medium at 28 °C for 4 days, the diameter of the SZ01 strain colony reaches 4.7 to 5.6 cm. The mycelium appears pubescent. The colony is white in the initial stage and gradually turns green in the later stage, with regular edges (Figs. 1a, b). Scanning electron microscopy revealed that vegetative hyphae of strain SZ01 were branched and interwoven, bearing a slightly rough surface (Fig. 1c). The conidia clustered on specialized conidiogenous structures; individual conidia were subglobose to ellipsoidal, measuring approximately 2–3 μm in diameter, and displayed a similarly rough surface texture (Fig. 1d). Combined with ITS and RPB2 sequence analysis (Fig. S2), strain SZ01 was preliminarily identified as *Trichoderma compactum.*

### Determination of the halotolerance threshold of strain SZ01

Strain SZ01 exhibited commendable salt tolerance, growing well on a medium containing 9% NaCl. As the salinity increases, microbial growth will be inhibited to varying degrees (Table 1). All plates were incubated at a constant temperature of 28 °C for 14 days and photographed simultaneously for observation (Fig. S3). In the salt-free control group (0% NaCl), the strain grew vigorously and produced abundant green conidia. Upon initial exposure to high-salinity conditions, growth was delayed in a salt-concentration-dependent manner. Specifically, strain SZ01 initiated growth on plates supplemented with 5%, 6%, and 7% NaCl by day 2, whereas growth on the 8% NaCl medium was delayed until day 5. On plates supplemented with 9% NaCl, visible growth did not appear until day 14. Under sustained high-salt conditions, colony expansion was restricted: on 8% NaCl plates, colony diameter plateaued at 5 cm, whereas on 9% NaCl plate, growth ceased at a diameter of only 1.5 cm.

**Table 1:** Growth of *T. compactum* SZ01 under different salt concentrations

NaCl Concentration (%)						
0%	5%	6%	7%	8%	9%	10%
+++	++	++	++	+	+	-

### Determination of the plant-growth-promoting traits of strain SZ01

**Fig. 2:**
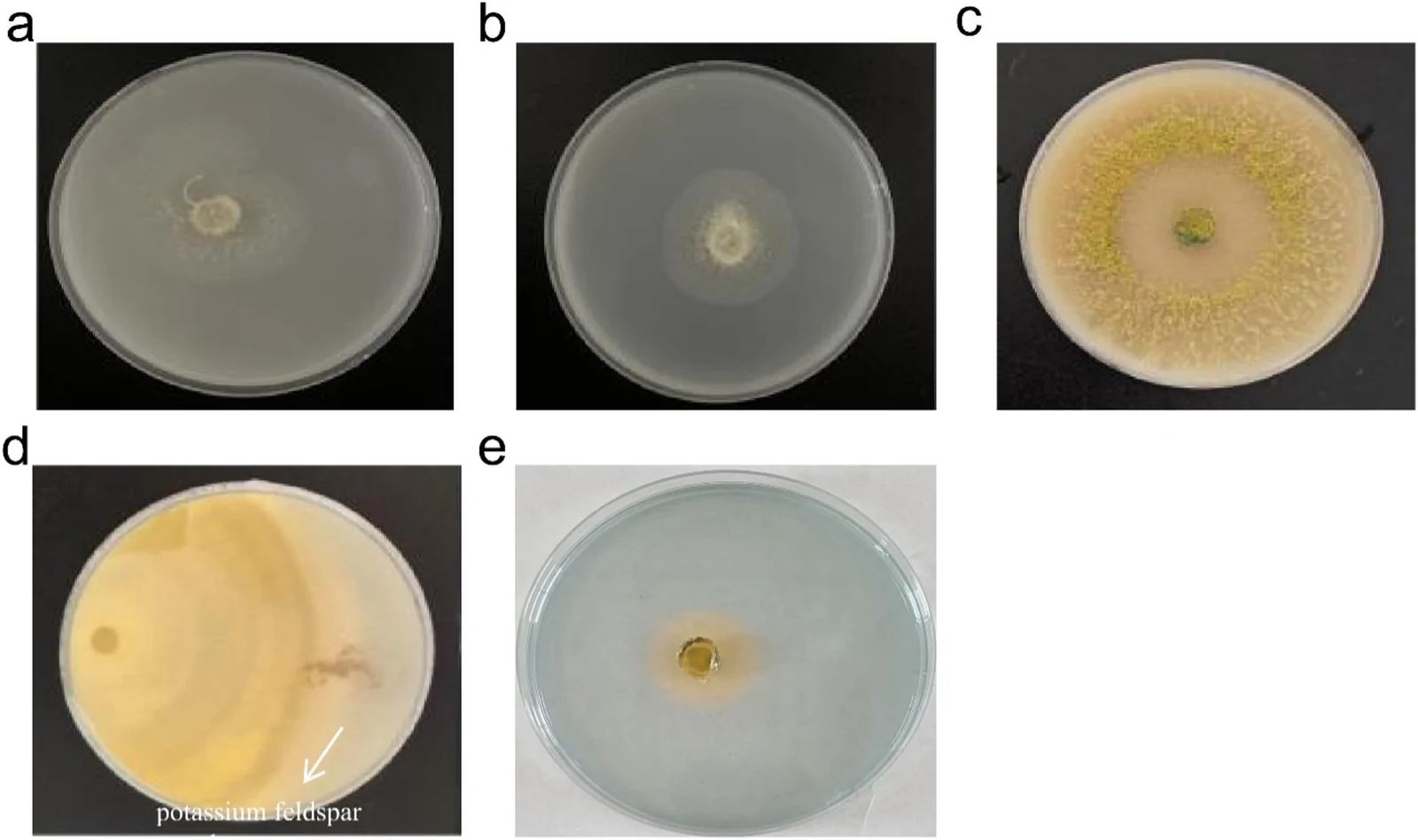
Plant-growth-promoting traits in *T. compactum* SZ01. **a** Organic phosphorus solubilization; (**b**) Inorganic phosphorus solubilization; (**c**) Nitrogen fixation; (**d**) Potassium solubilization; (**e**) Siderophore detection via CAS assay

After inoculating SZ01 onto organic phosphorus and inorganic phosphorus media and incubating for 5 days, no transparent zones were observed (Figs. 2a, b), indicating that the strain does not possess phosphorus-solubilizing ability. In contrast, after 5 days of cultivation on Ashby’s medium, the strain grew normally (Fig. 2c), demonstrating its nitrogen-fixing ability. For the potassium-solubilizing ability assay, the strain was inoculated onto PDA plates with a small amount of sterilized K-feldspar powder spread on one side; after incubation at 28℃ for 3 days, obvious chemotaxis of the mycelium toward the potassium-containing mineral powder was observed on the plates (Fig. 2d), suggesting that the strain has potassium-solubilizing ability. Following 5 days of culture on CAS detection medium, a characteristic orange-yellow secretion zone appeared in the medium (Fig. 2e), indicating that the strain is capable of producing siderophores. The results of the strain’s functional characterization showed that SZ01 possesses nitrogen-fixing, potassium-solubilizing, and siderophore-producing capabilities.

### Evaluation of strain SZ01 in alleviating salt stress and promoting growth in *C. chinensis* seedlings

#### Impact of microbial inoculation on growth parameters under salinity stress

**Fig. 3:**
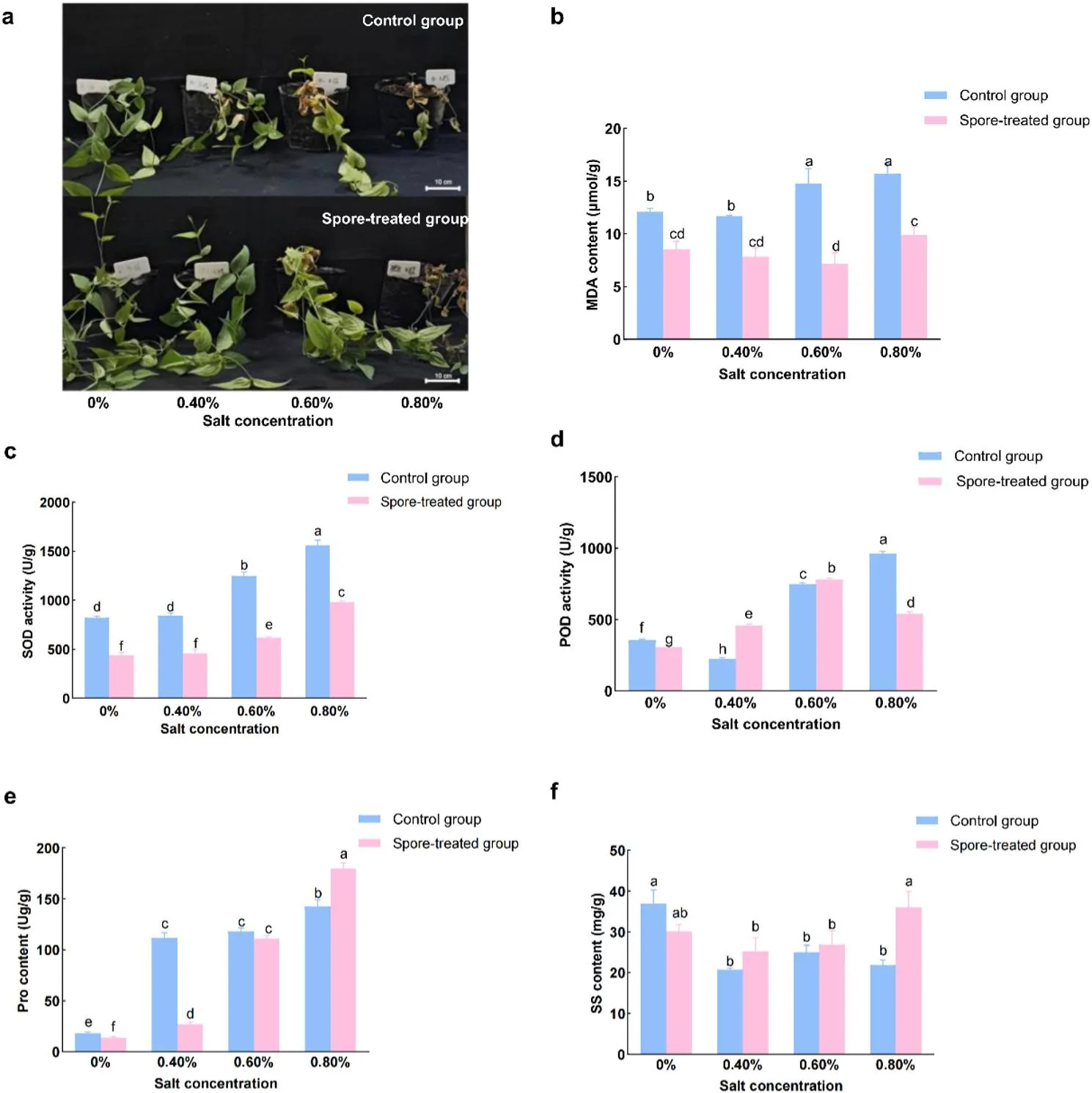
Effects of *T. compactum* SZ01 inoculation on plant growth and physiological characteristics under salt stress. **a** Phenotypes of plants under salt stress; (**b**) MDA content; (**c**) SOD activity; (**d**) POD activity; (**e**) Pro content; (**f**) SS content. All experiments were conducted with three biological replicates. Different letters indicate a significant difference (*p* < 0.05)

Under non-saline control conditions, the fresh and dry weights of *C. chinensis* roots in the spore-treated group were significantly higher than those in the water control group. Across three salt-stress treatments, *C. chinensis* supplemented with the spore suspension exhibited better growth performance (Fig. 3a), higher root fresh weight (RFW) and root dry weight (RDW) compared to control group (Table 2). At 0.40% salt, the fresh and dry weights of *C. chinensis* roots in the spore-treated group increased by 48.45% and 14.02%, respectively, relative to control group. As the salt concentration increased to 0.60%, the corresponding increases reached 159.76% and 117.68%. When the salt concentration reached 0.80%, the fresh and dry weights were further elevated by 205.57% and 157.74%, respectively, compared with the control.

**Table 2:** Effects of *T. compactum* SZ01 on the fresh and dry weights of *C. chinensis* roots under different salt concentrations

Salt treatment		RFW (g)	RDW (g)
0%	control group	7.09 ± 0.12b	1.39 ± 0.07b
	spore-treated group	11.27 ± 0.38a	2.65 ± 0.08a
0.40%	control group	2.62 ± 0.19b	0.64 ± 0.01b
	spore-treated group	3.89 ± 0.09a	0.72 ± 0.02a
0.60%	control group	2.78 ± 0.05b	0.61 ± 0.03b
	spore-treated group	7.20 ± 0.16a	1.32 ± 0.49a
0.80%	control group	1.86 ± 0.08b	0.34 ± 0.03b
	spore-treated group	5.67 ± 0.26a	0.86 ± 0.05a

#### Influence of microbial inoculation on MDA content under salinity stress

Salinity stress significantly induced lipid peroxidation, as evidenced by the marked elevation of MDA content in *C. chinensis* tissues. Conversely, inoculation with SZ01 spores effectively mitigated this oxidative damage, alleviating the phytotoxic effects of salt stress (Fig. 3b). When salt concentration increased from 0% to 0.80%, MDA content in *C. chinensis* leaves ranged from 11.66 to 15.69 µmol·g^− 1^ FW in the control group and from 7.18 to 9.93 µmol·g^− 1^ FW in the spore-treated group. Specifically, under 0.40% NaCl stress, the MDA content in the water control group showed no significant difference compared to the non-salt control. At 0.60% NaCl, MDA content in the water control group increased significantly (*P* < 0.05) but remained non-significant relative to the 0.80% NaCl group. In contrast, the spore-treated group exhibited a non-significant upward trend in MDA accumulation until 0.60% NaCl. However, MDA content was significantly reduced in the spore-treated group compared to the control group (*P* < 0.05).

#### Influence of microbial inoculation on SOD activity under salinity stress

Under salt stress, SOD activity in *C. chinensis* exhibited a significant dose-dependent increase with increasing NaCl concentrations. When NaCl concentrations increased to 0.60% and 0.80%, SOD activities were obviously increased compared to the non-salt control. SOD activities exhibited comparable trends between the spore-treated and control groups. When NaCl concentrations exceeded 0.40%, the stimulatory effect of SZ01 on SOD activity in *C. chinensis* progressively intensified with increasing salinity (Fig. 3c). However, SOD activities in the spore-treated group were lower than those in the control group.

#### Influence of microbial inoculation on POD activity under salinity stress

POD activity in leaves showed a biphasic response to high-salt stress, and then increasing with increasing NaCl concentrations in control group. In the control group, POD activity at 0.60% and 0.80% NaCl concentrations was 109.53% and 170.42% higher than that in the 0% NaCl group, respectively. The spore-treated group maintained relatively low POD activity under non-salt conditions. Following salt stress, POD activity in the group treated with spore was enhanced by 48.49%, 154.25%, and 79.44% under 0.40%, 0.60%, and 0.80% NaCl treatments, respectively, compared to the non-salt control. At 0.40% and 0.60% NaCl, the spore-inoculated group exhibited a significant enhancement in POD activity, surpassing the control by 37.51% and 4.63%, respectively (Fig. 3d). These results indicate that under salt stress, SZ01 spore inoculation initially enhanced POD activity in *C. chinensis* in a dose-dependent manner, but the promotive effect diminished with increasing salinity.

#### Impact of microbial inoculation on osmolyte accumulation under salinity stress

Proline is a crucial compound in plants to prevent osmotic damage. Upon exposure to biotic and abiotic stresses, proline accumulates in plant tissues significantly. With increasing salt concentrations, Pro contents in both treatment groups exhibited significant elevations (Fig. 3e). In the control group, Pro contents under three salt concentration treatments increased by 513.82%, 549.80%, and 682.44%, respectively, compared to the non-salt control. In the spore-treated group, Pro content increased by 95.85%, 696.82%, and 1188.42%, respectively, relative to the non-salt control. The highest Pro accumulation was (179.83 µg/g) occurred in the 0.8% salt treatment of the spore group, representing a 26.29% enhancement over the control group under identical salt stress. These results indicated that SZ01 inoculation promoted Pro accumulation in *C. chinensis* leaves.

Soluble sugars play multifaceted roles in plant growth, development, and stress responses, with the functions being particularly critical under stress conditions. The trend of SS content changes in *C. chinensis* under salt stress differed from that of Pro content. In the non-salt control, spore inoculation did not significantly elevate SS levels. When salt concentrations ranged from 0.40% to 0.80%, the SS content variations were 25.26–36.04 mg·g⁻¹ in the spore-treated group and 20.73–24.97 mg·g⁻¹ in the control group. Specifically, at salt concentrations of 0.40%, 0.60%, and 0.80%, spore treatments enhanced SS content by 21.86%, 7.76%, and 64.99%, respectively, with a statistically significant difference observed at 0.80% salt concentration (*P* < 0.05) (Fig. 3f). The findings demonstrate that SZ01 spore inoculation promotes SS accumulation in *C. chinensis* under salt stress, with the most pronounced effect observed at 0.8% salt concentration.

### Transcriptome sequencing

#### Principal component analysis

**Fig. 4:**
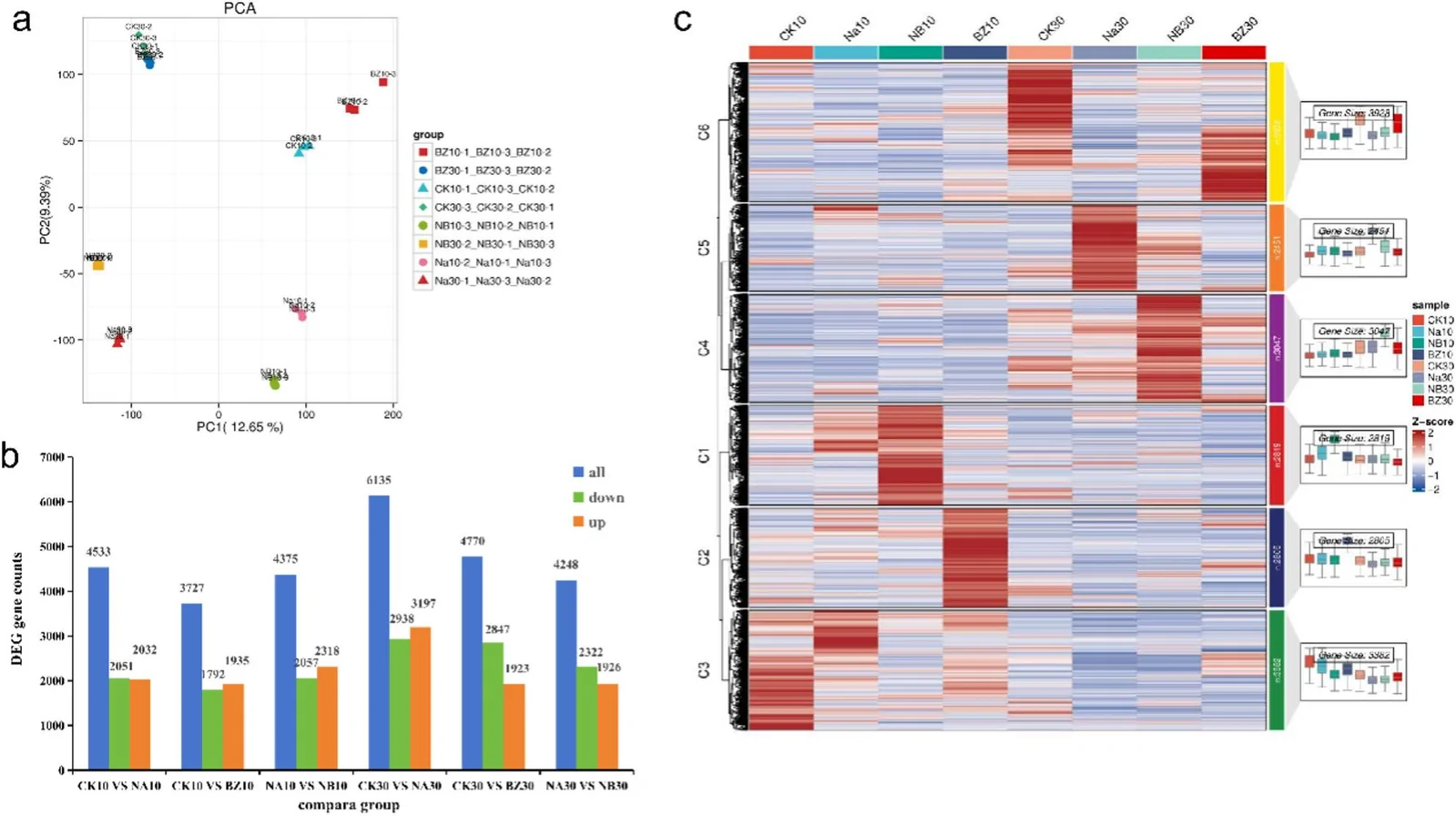
PCA, DEG statistics, and gene expression heatmap of *C. chinensis* under different treatments. **a** PCA of gene expression profiles (PC1: 12.65%, PC2: 9.39%); symbols represent different treatment groups. **b** DEG statistics (Fold Change ≥ 2, FDR < 0.01) in pairwise comparisons; "all/up/down" = total/up-regulated/down-regulated DEGs. **c** Z-score-normalized gene expression heatmap; rows = gene clusters (C1-C6), columns = samples; color = expression deviation. Note: CK = water treatment; NA= 0.60% NaCl treatment; NB = 0.60% NaCl + 10⁸ CFU·mL⁻¹ SZ01 spores treatment; BZ = water + 10⁸ CFU·mL⁻¹ SZ01 spores treatment

The results of Principal Component Analysis (PCA) showed that the biological replicates of all samples exhibited a tight clustering pattern in the two-dimensional space (Fig. 4a), indicating high reproducibility of the experiment and reliability of the data. Although the first principal component (PC1) explained only 12.65% of the total variance, the samples still displayed a gradient distribution trend related to treatment conditions along the PC1 axis, suggesting that different treatments might be the main factor driving differences in gene expression. In contrast, the second principal component (PC2) contributed less to the variance (9.39%) and did not show obvious separation between groups. This analysis verified the stability of the experimental operation and provided a quality basis for subsequent differential expression studies.

#### Identification of DEGs in *C. chinensis* under different treatments

We analyzed the transcriptomic response to salt stress by performing RNA-seq on leaf samples from four treatment groups (CK, NA, NB, BZ) at 10- and 30-day time points, identifying DEGs with a |log₂FC| ≥ 1 and FDR < 0.01 threshold (Fig. 4b). The number of DEGs generally increased from 10 to 30 days across all comparisons. This trend was most pronounced in the CK vs. NA group, where the DEG count jumped from 4,375 to 6,135. For the CK vs. BZ group, the total DEG number increased from 3,727 to 4,770; the number of up-regulated genes barely changed, while down-regulated genes rose by nearly 60%. For the NA vs. NB group, the DEG count decreased by only 2.90% with no obvious overall change, but the number of down-regulated genes increased by 12.88%, whereas the number of up-regulated genes decreased by 16.91%.

#### Clustering analysis of expression patterns of DEGs

We clustered all DEGs based on their expression patterns, revealing six distinct modules (C1–C6) of varying sizes (Fig. 4c). The modules showed clear, sample-specific expression profiles. For example, genes in module C3 were highly active in the CK10, NA10, and BZ10 samples but were repressed in NA30 and NB30. Module C2 presented a mirror image of this, with a surge of activity in BZ10 and strong repression in the 30-day CK, BZ and NA samples.

#### GO functional annotation of DEGs

**Fig. 5:**
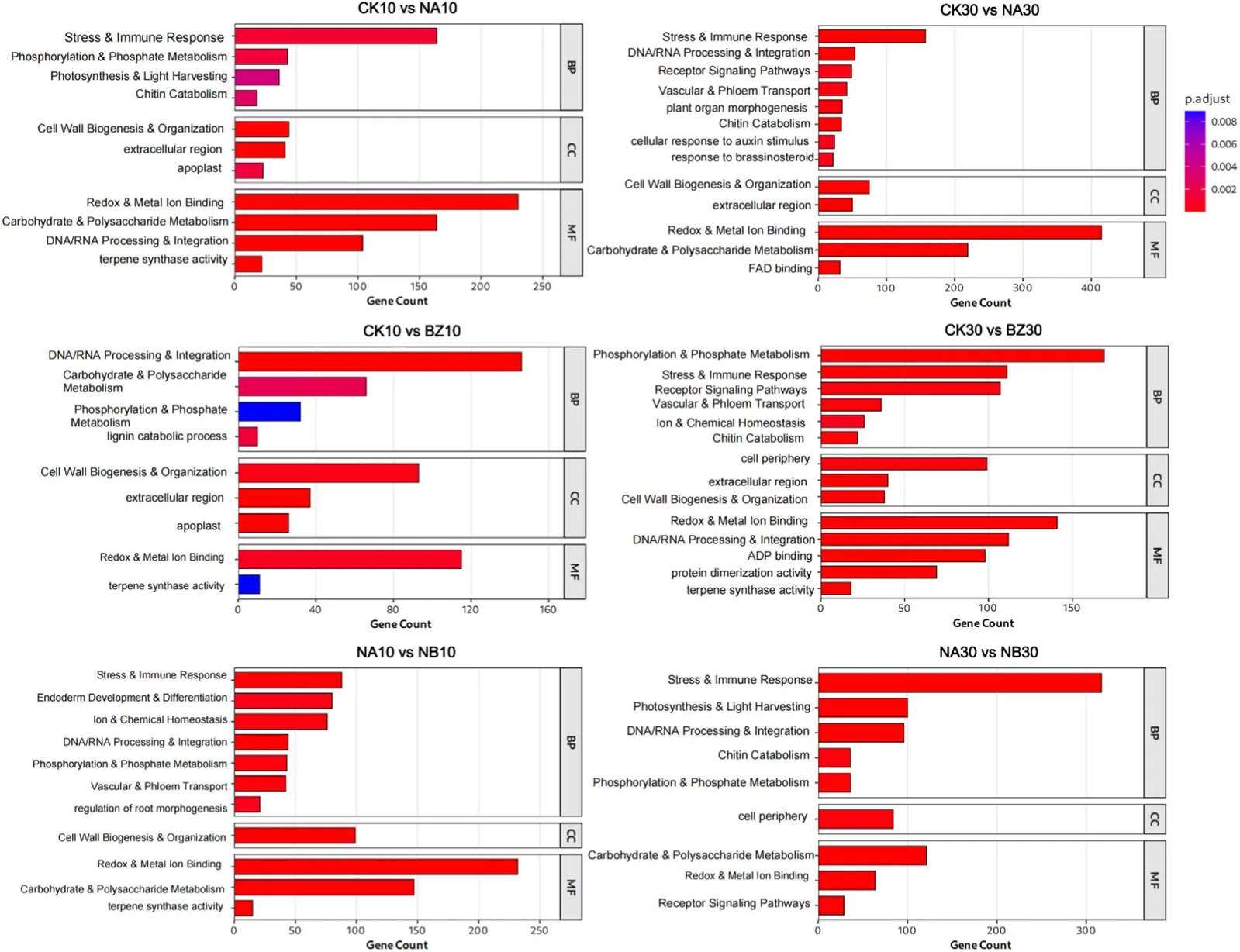
GO enrichment analysis of DEGs across multiple group comparisons at 10 and 30 days

To systematically analyze the functional distribution characteristics of differentially expressed genes under salt stress and treatment with salt-tolerant growth-promoting bacteria, a GO functional enrichment analysis was performed on the differentially expressed genes. All enriched terms were classified into three major categories: biological processes (BP), cellular components (CC), and molecular functions (MF) (Fig. 5).

In the CK10 vs. NA10 comparison, the core BP pathway was “Stress & Immune Response,” with enrichment in the “Phosphorylation & Phosphate Metabolism” and “Photosynthesis & Light Harvesting” pathways; CC was primarily enriched in “Cell Wall Biogenesis & Organization,” “extracellular region,” and “apoplast” pathways; MF is dominated by “Redox & Metal Ion Binding” and “Carbohydrate & Polysaccharide Metabolism” pathways; In the CK30 vs. NA30 comparison, the BP-enriched pathway “Photosynthesis & Light Harvesting” was not enriched, while entries such as “DNA/RNA Processing & Integration,” “Receptor Signaling Pathways,” and “Vascular & Phloem Transport” increased; there were no significant changes in the CC-enriched entries; in MF, the number of genes enriched in “Redox & Metal Ion Binding” increased substantially, with “FAD binding” added. The results indicate that salt stress inhibits the plant’s photosynthesis-related functions, forcing the plant to maintain a prolonged state of stress and immune response, which clearly demonstrates the damaging effects of salt stress on plant growth and metabolism.

In the CK10 vs. BZ10 comparison, BP showed the greatest enrichment in “DNA/RNA Processing & Integration”, along with enrichment in “Carbohydrate & Polysaccharide Metabolism”, “Phosphorylation & Phosphate Metabolism”, and “lignin catabolic processes”; CC was primarily enriched in “Cell Wall Biogenesis & Organization”, “the extracellular region”, and “the apoplast”; MF’s core category was “Redox & Metal Ion Binding,” accompanied by enrichment in “terpene synthase activity”; Comparing CK30 and BZ30, there was a significant shift in the BP categories, with “Phosphorylation & Phosphate Metabolism” becoming the category accounting for the highest proportion. New categories “Stress & Immune Response,” “Receptor Signaling Pathways,” “Vascular & Phloem Transport,” and “Ion & Chemical Homeostasis” were included. CC newly added “cell periphery,” while the number of enriched genes in the existing entries remained unchanged; MF introduced “ADP binding” and “protein dimerization activity,” and the number of enriched genes in “Redox & Metal Ion Binding” and “DNA/RNA Processing & Integration” increased significantly. The findings indicated that under salt-free conditions, strain SZ01 could sequentially activate growth and development-related functional categories such as “DNA/RNA Processing & Integration” (nucleic acid proliferation), “Cell Wall Biogenesis & Organization” (cell wall construction), and “Vascular & Phloem Transport” (nutrient transport), demonstrating the molecular potential to regulate normal plant growth.

Comparing NA10 and NB10, the most highly enriched entry in the BP category was “Stress & Immune Response”, followed by “Endoderm Development & Differentiation”, “Ion & Chemical Homeostasis”, “DNA/RNA Processing & Integration”, “Phosphorylation & Phosphate Metabolism”, “Vascular & Phloem Transport”, and “Regulation of Root Morphogenesis”; In the CC category, only “Cell Wall Biogenesis & Organization” was significantly enriched; within the MF category, “Redox & Metal Ion Binding” had the highest number of enriched genes, followed by “Carbohydrate & Polysaccharide Metabolism,” accompanied by a small degree of enrichment in “Terpene Synthase Activity.” In the NA30 vs. NB30 comparison, the number of enriched genes in “Stress & Immune Response” increased significantly, becoming the most highly enriched category among the BP supercategories. Additionally, “Photosynthesis & Light Harvesting” and “Chitin Catabolism” were newly enriched, while “DNA/RNA Processing & Integration” remained enriched; and the previously enriched categories related to endodermis development and root morphogenesis were no longer significantly enriched; While CC retained only the “Cell Periphery” category, MF’s dominant category shifted to “Carbohydrate & Polysaccharide Metabolism,” the enrichment level of “Redox & Metal Ion Binding” decreased, and “Receptor Signaling Pathways” was newly added. The results indicate that inoculation with SZ01 under salt stress conditions can restore significant enrichment of the “Photosynthesis & Light Harvesting” category and enhance the processes of “Ion & Chemical Homeostasis” and “Stress & Immune Response,” effectively alleviating the growth inhibition caused by salt stress.

### KEGG pathway distribution of DEGs

To further elucidate the core metabolic pathways involved in the differentially expressed genes under salt stress and strain treatment, a KEGG pathway enrichment analysis was performed on the differentially expressed genes in each group (Fig. 5).

**Fig. 6:**
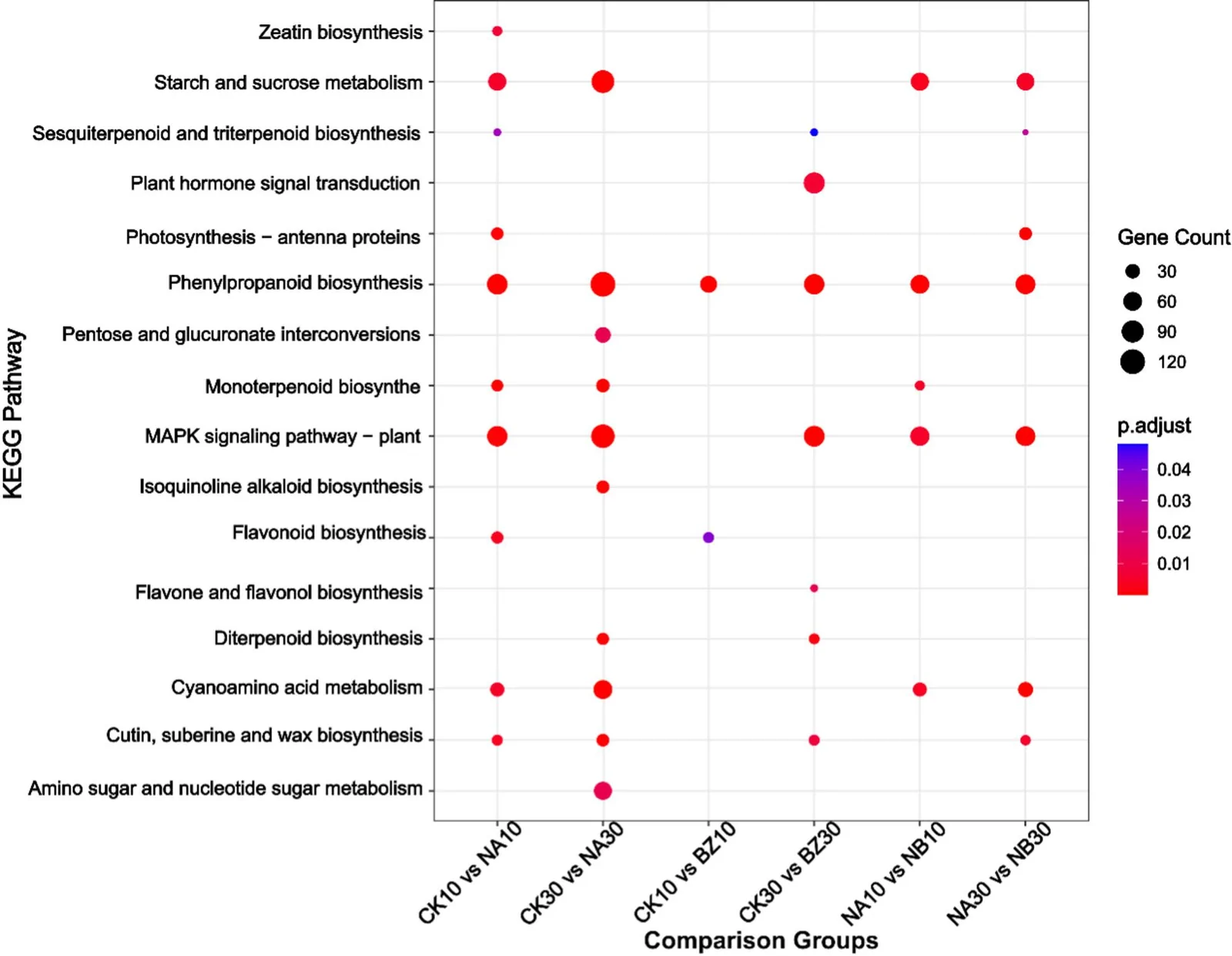
KEGG pathway enrichment analysis of DEGs across multiple group comparisons at 10 and 30 days

In the CK10 vs. NA10 comparison, the following pathways, “MAPK signaling pathway-plant”, “Starch and sucrose metabolism”, “Phenylpropanoid biosynthesis”, and “Cutin, suberin, and wax biosynthesis”, were significantly enriched. Simultaneously, the pathways for “Zeatin biosynthesis”, “Photosynthesis-antenna proteins”, “Monoterpenoid biosynthesis”, “Flavonol biosynthesis”, and “Sesquiterpenoid and triterpenoid biosynthesis” were specifically activated. In the CK30 vs. NA30 comparison, pathways enriched under short-term salt stress, such as “Photosynthesis-antenna proteins”, “Flavonol biosynthesis”, and “Zeatin biosynthesis”, were no longer enriched, indicating that a long-term high-salt environment significantly inhibits the plant’s photosynthetic metabolic pathways. MAPK signaling pathway-plant, starch and sucrose metabolism, phenylpropanoid biosynthesis, cyanoamino acid metabolism, and cutin, suberin, and wax biosynthesis-remained significantly enriched; concurrently, this group exhibited multiple specifically enriched pathways, including amino sugar and nucleotide sugar metabolism, pentose and glucuronate interconversions, isoquinoline alkaloid biosynthesis, and diterpenoid biosynthesis. This indicated that long-term salt stress significantly reshaped the plant’s secondary metabolic pathways for carbohydrates and alkaloids, causing a massive diversion of carbon sources and amino acids toward stress-response metabolism, while nutrient supply deviated from the requirements for normal growth and development, ultimately leading to growth inhibition and exacerbated physiological damage (Fig. 6).

In CK10 vs. BZ10, the phenylpropanoid biosynthesis pathway was significantly enriched; this group specifically showed enrichment in flavonoid biosynthesis, while photosynthesis and zeatin-related pathways were not significantly enriched. In CK30 vs. BZ30 comparison, this group exhibited a uniquely highly enriched pathway-plant hormone signal transduction-along with significant enrichment of phenylpropanoid biosynthesis and the MAPK signaling pathway in plants. Additionally, new pathways were identified, including flavone and flavonol biosynthesis, diterpenoid biosynthesis, cutin, suberin, and wax biosynthesis, sesquiterpenoid and triterpenoid biosynthesis. Overall, the profile was characterized primarily by hormone signal transduction and the regulation of flavonoid secondary metabolism. The results indicated that under non-stress conditions, SZ01 exerted growth-promoting effects primarily by regulating growth-related metabolic pathways and did not induce metabolic disruptions associated with stress.

In NA10 vs. NB10, “Starch and sucrose metabolism”, “Phenylpropanoid biosynthesis”, “MAPK signaling pathway-plant”, “Cyanoamino acid metabolism”, “Monoterpenoid biosynthesis” all exhibited significant enrichment. In the NA30 vs. NB30 comparison, the pathways “Cutin, suberin, and wax biosynthesis”, “Photosynthesis-antenna proteins” and “Sesquiterpenoid and triterpenoid biosynthesis” were newly identified as significantly enriched; “Starch and sucrose metabolism”, “MAPK signaling pathway-plant”, and “Phenylpropanoid biosynthesis” remained significantly enriched. These results indicated that the inoculated strain SZ01 exerted a time-dependent regulatory effect under salt stress conditions: short-term inoculation maintained basic stress responses and homeostasis in carbohydrate metabolism; long-term treatment restored the enrichment of pathways related to light capture in photosynthesis and synergistically activated terpenoid stress-resistant secondary metabolism, thereby mitigating the inhibitory effects of high salinity on plant growth and metabolism.

### Analysis of secondary metabolites produced by halo tolerance strain SZ01 under high salt stress

**Fig. 7:**
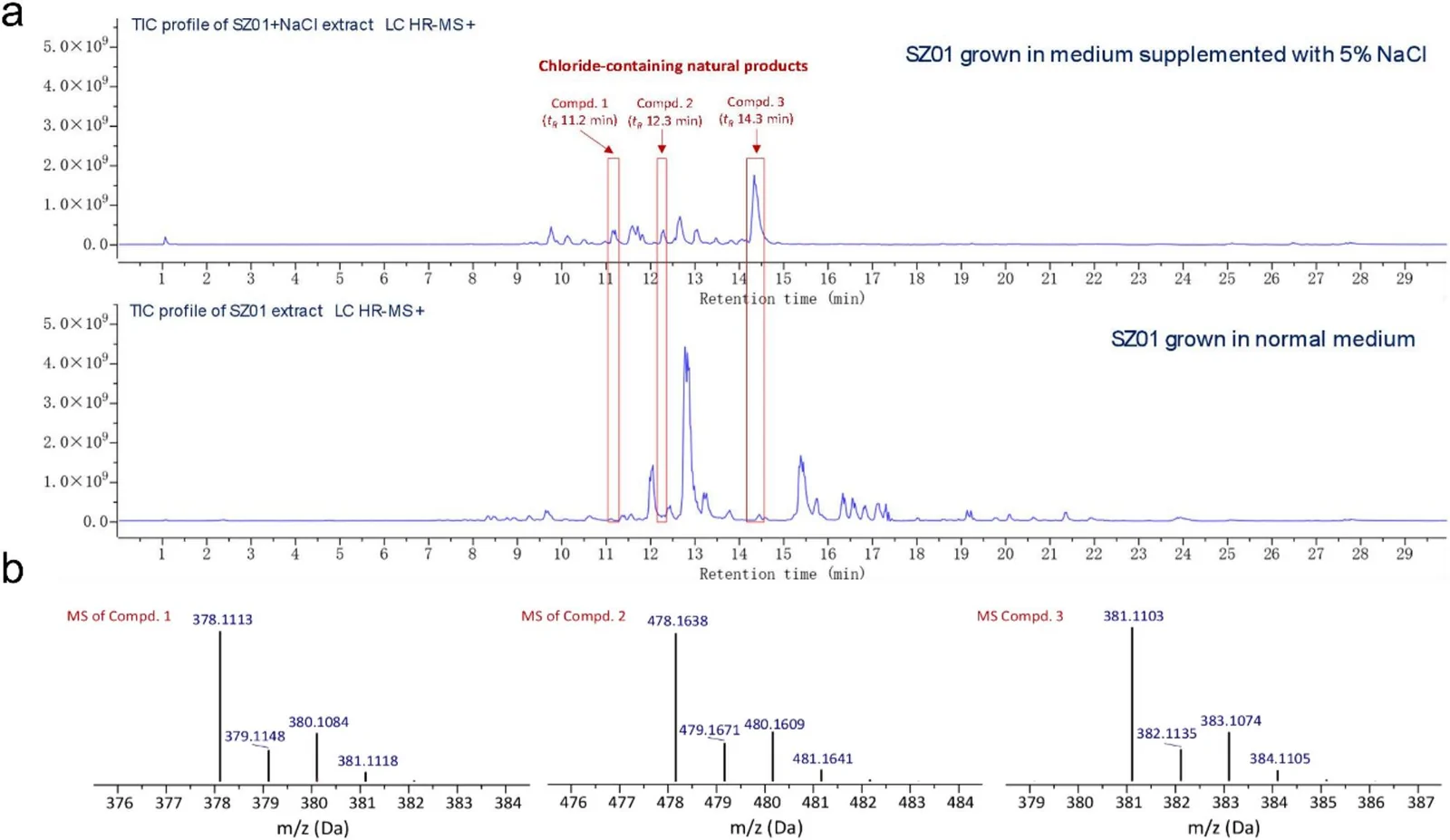
Comparison of secondary metabolites produced by strain SZ01 under normal and salt stress conditions by LC-MS. **a** Total ion chromatogram (TIC) profiles of SZ01 culture extracts. The major accumulated peaks were highlighted with red squares. **b** High resolution mass spectra of compounds 1, 2, and 3. The peak abundance ratios of m/z 378 to m/z 380, m/z 478 to m/z 480, and m/z 381 to m/z 383 were all 3:1

Interactions between rhizosphere microorganisms and plants constitute an important and rapidly advancing research area. Increasing evidence indicates that natural products function as key signaling molecules in such interactions, substantially deepening our understanding of chemical ecological. For example, inoculation with arbuscular mycorrhizal fungi has been shown to significantly enhance legume nodulation by stimulating the production of flavonoids and phenolic acids. To investigate the potential contribution of secondary metabolites to the growth-promoting effects of strain SZ01 on *C. chinensis*, we performed comparative chemical analyses of strain SZ01 cultured in standard medium and medium supplemented with 5% NaCl (Fig. 7). High-resolution liquid chromatography–mass spectrometry (LC–HR-MS) revealed a pronounced accumulation of compounds under high-salt conditions relative to the non-salt control (Fig. 7a). Detailed MS analysis of three major accumulated compounds displayed a characteristic chlorine isotope pattern, with an M: (M + 2) peak ratio of 3:1, suggesting the production of chlorinated molecules by strain SZ01 in response to salt stress (Fig. 7b). The biological functions of these compounds remain to be elucidated and are the subject of ongoing investigation.

## Discussion

**Fig. 8:**
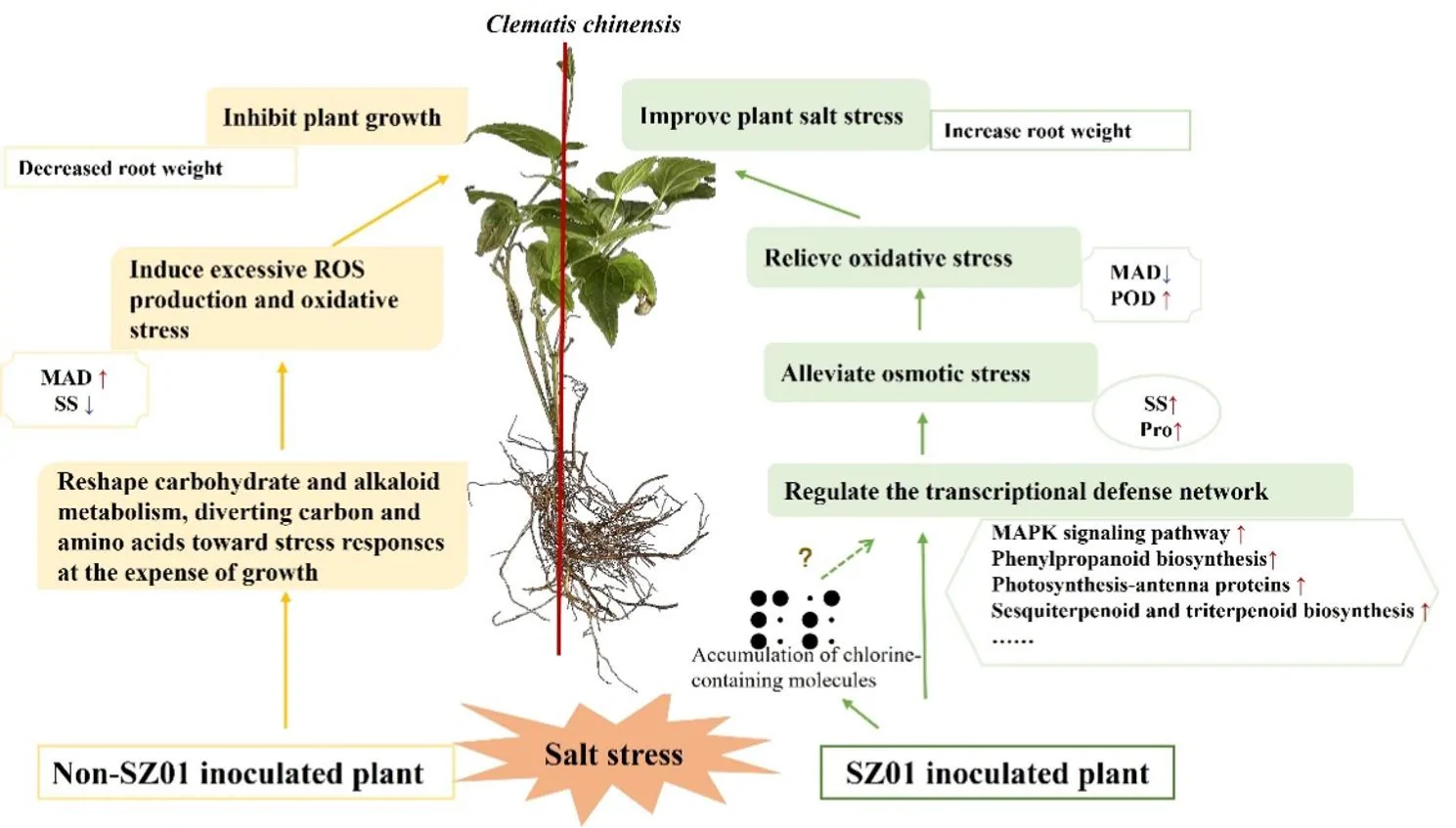
Schematic diagram of the mechanism by which SZ01 promotes salt tolerance in *C. chinensis*

As global soil salinization intensifies, the exploration of salt-tolerant, plant growth-promoting microbes is critical for sustainable agriculture [37]. In this study, we isolated a *T. compactum* strain (designated SZ01) from saline soil. Characterization of its extreme salt tolerance and PGP traits confirmed its potential as a plant biostimulant under saline stress. Furthermore, pot experiments were performed to evaluate the effects of SZ01 on the physiological and biochemical properties of *C. chinensis* under salt stress. Combined with transcriptome sequencing, this study elucidated the molecular mechanism by which SZ01 enhances plant salt tolerance and promotes growth, providing preliminary insights into its underlying modes of action (Fig. 8).

A strain’s degree of halotolerance is a primary determinant of its ecological fitness in saline environments. In this study, SZ01 exhibited robust growth at moderate salinities (5–7% NaCl). Although growth of the strain was inhibited at 8% NaCl, it maintained viability at concentrations up to 9%. Although growth was suppressed at 8% NaCl, the strain remained viable at a threshold of 9% NaCl. This remarkable resilience indicates a strong competitive advantage for colonization of salt-affected soils. These results are in agreement with findings by Wingfield et al., who documented that *Penicillium citrinum* (NM-3) and *Fusarium equiseti* (NM-8) from Thai salt pans also displayed exceptional haloadaptation, thriving across a broad 3–15% NaCl gradient [38].

Research has confirmed that plant growth-promoting fungi play a vital role in enhancing plant stress resilience and productivity under saline conditions. These fungi employ multifaceted mechanisms to concurrently promote plant growth and bolster salt tolerance [39]. This functional paradigm is strongly supported by findings from Al-Sissi et al. [40], who isolated the halotolerant fungal strains *Alternaria* sp. AS1 and *Aspergillus terreus* B4 from the rhizosphere of the halophyte *Atriplex nummularia*. These strains significantly improve plant nutrient acquisition efficiency through a multipronged strategy, including siderophore secretion and solubilization of poorly available soil phosphorus, thereby effectively mitigating the adverse impacts of salt stress and comprehensively facilitating plant growth.

Our morphological and physiological assays demonstrate that strain SZ01 significantly enhances the growth and salt stress tolerance of *C. chinensis*, marking it as a promising candidate for biofertilizer development. SZ01 inoculation substantially enhancing root biomass even under non-saline conditions—fresh biomass increased by 59.04% and dry biomass by 91.17% relative to the control. Intriguingly, under increasing salinity, root biomass displayed a biphasic response, peaking at 0.6% NaCl before declining. This adaptive root response aligns with previous observations, such as the trend reported by Liu et al. [41] for *Trichoderma longibrachiatum* HL167 enhancing cowpea root growth under saline conditions.

MDA content exhibited a positive correlation with salt concentration across all treatments, while the SZ01-inoculated group displayed significantly lower MDA levels than the control, with a 51.49% reduction under 0.6% NaCl stress. This finding suggests that SZ01 application ameliorates salt stress-induced cellular damage in *C. chinensis*. Similar results were reported by Chauhan et al. [42]: the halotolerant plant growth-promoting fungus *Aspergillus terreus* CR7 reduced MDA content in *Vigna radiata* by 43.45% under severe salt stress (250 mM NaCl), alongside a 51.50% reduction in electrolyte leakage. These effects collectively mitigate salt stress-triggered lipid peroxidation and membrane disruption, thereby alleviating plant injury.

At the molecular and physiological level, salt stress elicits excessive Na⁺ uptake in plant roots, leading to ionic imbalance, metabolic perturbation, and subsequent ROS over accumulation. Elevated activities of SOD and POD represent a passive defensive response to scavenge excess ROS, with higher enzyme activities indicative of greater oxidative stress intensity and defense burden [43]. In the control group, SOD and POD activities in *C. chinensis* increased with rising salt concentrations, reflecting pronounced ROS accumulation and oxidative stress. In the spore-treated group, POD activity peaked at 781.36 U/g under 0.60% NaCl, representing a 4.63% increase compared to the control group. However, POD activity decreases significantly when the salt concentration reached 0.80%. In contrast, under identical salt treatments, SZ01-inoculated significantly reduced SOD activity compared to the control group, which was similar to the Ning Z et al. (2024) results [44]. A possible explanation is that the inoculated microorganisms formed a biofilm on the roots of the *C. chinensis*, reducing the roots’uptake of Na^+^, thereby alleviating the stress response. Unlike the consistent decline in SOD activity observed across all salinity levels, POD activity in SZ01-inoculated plants was significantly higher than that in the control group under 0.4% and 0.6% NaCl treatments. This differential response pattern aligns with the findings of Hu et al. (2025), who reported that *Trichoderma asperellum* 22,043 exerts distinct regulatory effects on SOD and POD under salt stress [45].

Pro and SS function as key osmotic regulators, whose accumulation facilitates water uptake and sustains cellular homeostasis under salt stress [46]. Under 0.40% NaCl stress, Pro content in the water control group increased by 513.82%, whereas the SZ01-inoculated group exhibited a much milder elevation (95.85%), potentially reflecting reduced salt sensitivity in inoculated plants. Pro content peaked in both groups at 0.80% NaCl, with the inoculated group displaying a 26.29% higher Pro level than the control. Given that enhanced stress tolerance is typically associated with elevated Pro accumulation [47], these data confirm that SZ01 inoculation enhances the stress resilience of *C. chinensis*. SS content in the water control group decreased as salt concentration increased from 0% to 0.40%, likely due to the catabolism of stored SS to fuel osmotic adjustment and metabolic processes during the early stages of salt stress. In contrast, SS levels in the SZ01-inoculated group were consistently higher than those in the control under 0.40%–0.80% NaCl treatments, with a significant difference observed at 0.80% NaCl. This indicates that SZ01 mediates the enhanced synthesis of osmoprotectants in *C. chinensis*.

Collectively, our findings demonstrate that SZ01 inoculation modulates a suite of physiological traits in *C. chinensis* under salt stress, thereby mitigating salt-induced damage. These results confirm that SZ01 enhances plant growth and salt tolerance, validating its dual role as a growth-promoting and halotolerant microbial agent. However, the direct evidence of successful root establishment remains to be elucidated. In subsequent research, we will conduct in-depth investigations into the colonization dynamics, translocation patterns, and molecular mechanisms governing the plant–microbe interaction between SZ01 and *C. chinensis*.

To elucidate the molecular mechanisms by which strain SZ01 alleviates salt stress and promotes plant growth, we investigated the transcriptional landscape of *C. chinensis* leaves under salt stress via RNA-Seq.

Initially, salt stress (CK vs. NA) induced canonical defensive transcriptional reprogramming in *C. chinensis*. A substantial number of differentially expressed genes (DEGs) were significantly up regulated and were predominantly enriched in functional categories such as “metabolic process”, “binding”, and “response to stimulus”, as well as key KEGG pathways including phenylpropanoid biosynthesis, plant MAPK signaling pathway, starch and sucrose metabolism, photosynthesis-antenna proteins, zeatin biosynthesis and terpenoid biosynthesis pathways. Mechanistically, the upregulation of metabolism-related genes provides a physiological basis for plant salt tolerance by promoting the synthesis of compatible solutes and reprogramming metabolic pathways. Meanwhile, genes involved in “response to stimuli” directly participate in the perception and transduction of salt stress signals. Downstream, activation of the phenylpropanoid biosynthesis pathway—which was persistently enriched under both 10 d and 30 d salt stress—exerts a dual protective effect: on one hand, it scavenges stress-induced ROS via antioxidant synthesis, alleviating oxidative damage; on the other hand, it promotes lignin deposition, reinforcing cell wall integrity and enhancing structural stability. The coordinated action of this multi-tiered network, from initial signal perception to terminal physiological and biochemical responses, constitutes the comprehensive transcriptional defense network of *C. chinensis* against salt stress. This observed expression pattern aligns perfectly with the established model of plant salt stress responses [48], reaffirming that a synergistic, multi-gene, and multi-pathway defensive transcriptional reprogramming strategy is a universal mechanism for plants to cope with salt stress.

Under SZ01 treatment alone (CK vs. BZ), we observed that the enrichment of upregulated genes in related pathways was stable, while down regulated genes significantly increased. This unique transcriptional pattern of “stable upregulation, expanded down regulation” forms a significant difference from the classic response pattern of “defensive broad upregulation” under salt stress. Further combining the temporal features of GO and KEGG, it can be known that this transcriptional pattern corresponds to a “precise optimization” of the plant’s basal metabolic network: in the early stage, SZ01 activated pathways like Phenylpropanoid biosynthesis and Starch and sucrose metabolism to strengthen structural defense and material reserves; in the later stage, by expanding the scope of downregulation, it suppressed redundant metabolic pathways that consume excess carbon and energy resources, while simultaneously focusing on core regulatory networks including Plant hormone signal transduction and the MAPK signaling pathway - plant. This process not only avoids the growth cost brought by stress induction, but also accumulates materials and energy needed for plant growth through metabolic reprogramming. This unique regulatory feature clearly indicates that the growth-promoting effect of SZ01 does not rely on activating typical salt stress defense pathways, but instead reshapes the plant metabolic network with an independent logic. This pattern is highly consistent with the classic growth-promotion model of plant rhizosphere-promoting bacteria — the latter precisely regulates host growth by directly producing plant hormones and optimizing nutrient absorption, which further confirms that the core mechanism of SZ01 achieving growth promotion is through direct intervention in the plant’s basal metabolism [49].

The key role of SZ01 is highlighted in the NA vs. NB group. Under salt stress, its regulation of the plant presents a core model of transcriptional network optimization, stress response convergence, and improved stress resistance efficiency. Although the total number of DEGs changed slightly (only a 2.90% reduction) from day 10 to day 30, the composition of DEGs was drastically reshaped: the number of upregulated genes declined while downregulated genes expanded, and the genes involved in redundant stress pathways were markedly reduced. KEGG enrichment analysis showed that SZ01 exerted obvious time-dependent regulation under salt stress. At day 10, five pathways including starch and sucrose metabolism, phenylpropanoid biosynthesis, plant MAPK signaling pathway, cyanoamino acid metabolism and monoterpenoid biosynthesis were significantly enriched to maintain plant carbon homeostasis and basic salt stress response. At day 30, all these pathways remained significantly enriched, with newly enriched “Cutin, suberine and wax biosynthesis” as well as “Sesquiterpenoid and triterpenoid biosynthesis”. Under long-term salinity, SZ01 continuously activated primary metabolism and various terpenoid secondary metabolic pathways to establish a multi-layer anti-stress metabolic network and relieve the inhibition of high salinity on plant growth and metabolism. On day 30, two root development pathways, “Endoderm Development & Differentiation” and regulation of root morphogenesis, were no longer enriched, while the enrichment of “Stress & Immune Response” was further enhanced, and the Photosynthesis & Light Harvesting pathway was newly enriched. Meanwhile, the core molecular function (MF) shifted from Redox & Metal Ion Binding to Carbohydrate & Polysaccharide Metabolism. Under short-term salt stress, plants consumed substantial transcriptional resources for root remodeling and intensive redox defense. At the later stress stage, strain SZ01 promoted plants to shut down root developmental programs and reallocate resources from persistent antioxidant responses to carbohydrate synthesis and photosynthetic carbon metabolism. Supported by stable energy supply, plants achieved long-term stress tolerance with greatly reduced unnecessary transcriptional consumption. These results indicate that SZ01 does not indiscriminately activate all biological processes under salt stress; instead, it remodels the original stress regulatory network of plants to optimize resource allocation, enabling plants to maintain cellular homeostasis at a lower transcriptional cost. This mechanism is consistent with the model of beneficial microbes enhancing host adaptability through “physiological optimization” [50].

Additionally, chemical-mediated interactions may contribute to the enhanced salt tolerance *C. chinensis* during symbiosis with strain SZ01. We observed significant differences in the secondary metabolite profiles of SZ01 under high salt versus normal condition. Notably, several accumulated LC-MS peaks exhibited characteristic chlorine-isotope patterns, suggesting the production of halogenated metabolites. Therefore, we propose that the exceptional salt tolerance of strain SZ01 may be partially attributed to the biosynthesis of halogenated compounds, which could substantially sequester environmental salt and thereby alleviate salt stress, ultimately facilitating the growth of its plant host. Moreover, those uncharacterized natural products may function as signal molecules involved in plant growth promotion, although their exact role remains to be elucidated. The participation of microorganism-derived compounds in plant-microbe interactions has been extensively documented and holds considerable promise for agricultural applications [51]. Further studies are underway to reveal the structure and bioactivity of these compounds.

In summary, we propose the following working model: *C. chinensis* adopts a resource-intensive “comprehensive defense” strategy under salt stress. The inoculation of SZ01, through a temporal regulation, transforms it into a more economical “pre-adaptation-optimization” strategy: pre-adjusting metabolism to benefit growth when there is no stress; and in a stressful environment, it reduces the plant’s stress load by optimizing signaling networks and metabolic processes, thereby using the saved resources to maintain growth, and ultimately achieving synergistic salt tolerance and growth promotion. These findings are consistent with previous reports demonstrating that *Bacillus subtilis* GB03 enhances plant stress tolerance through modulation of host gene expression [52]. Roots represent the key site of fungal–plant interaction. Although our current study included some preliminary observations of root responses, a comprehensive analysis of root physiological and molecular changes was not performed. We intend to address this in future research by systematically examining root development, ion transport, and fungal–root molecular crosstalk, thereby providing deeper insight into SZ01-mediated growth promotion under salt stress.

## Conclusion

*T. compactum* SZ01, isolated from the rhizosphere of *C.chinensis*, is a high salt-tolerant fungal strain capable of tolerating 9% NaCl concentration. Functional characterization showed that SZ01 exhibited potassium solubilization, nitrogen fixation potential, and siderophore production, although it lacked the ability to solubilize either organic or inorganic phosphorus. Pot experiments showed that inoculation with SZ01 spore suspension significantly increased the fresh and dry weights of subterranean tissues under salt stress, decreased the accumulation of MDA, and increased the contents of soluble sugar and proline. Additionally, transcriptomic analysis of *C. chinensis* leaves revealed that SZ01 treatment induced considerable transcriptional reprogramming, especially through MAPK and metabolic pathway regulation, improving stress-responsive gene expression. These results suggest that SZ01 could be an effective mitigation of salt-induced physiological damage, and the accumulation of chlorine compounds may aid in promoting plant growth.

## Supplementary Information

Supplementary Material 1: Figure S1-S4 and Table S1.

## Data Availability

Data supporting the findings of this work are available within the paper and its Supporting Information. The DNA sequences have been deposited in the National Center for Biotechnology Information (NCBI) GenBank under accession numbers PZ368428 and PZ387666. The transcriptome data have been deposited in the Genome Sequence Archive (GSA) of China National Center for Bioinformation (CNCB) under the accession number CRA069850, but are not yet publicly accessible due to privacy restrictions. Researchers interested in accessing the data may contact the corresponding author for further information.
