# Supplementary material for: Enhancement of growth and salt tolerance in Clematis chinensis by the plant growth- promoting fungus Trichoderma compactum SZ01
Source: BMC Plant Biol. 2026 Jul 31;26:1838. doi: 10.1186/s12870-026-09648-7 (PMC13715118; doi:10.1186/s12870-026-09648-7)
Supplement: Supplementary file 1 [file 12870_2026_9648_MOESM1_ESM.docx]

Table S1 Primer sequences of target genes and the reference gene Actin for qRT-PCR validation of transcriptome data

| Gene ID | Gene annotation | Primer sequence ( 5'-3') |
| --- | --- | --- |
| DN7936 | WRKY DNA -binding domain | F:TGCTGTCTTCAAGTTCGAGT  R:TGAGAGAAAATACCACCCCG |
| DN3981 | Sodium/hydrogen exchanger family | F:GTACACTGGAAAGCAACAGG  R:TGGGATTTGGCTTTGACAGA |
| DN5405 | Universal stress protein family | F:TAGTAGCCATAGACAGCGGA  R:AATATCCATCGCTGCCTTCT |
| DN2381 | Protein kinase domain | F:CACGAAGAATGGGAGGATCA  R:TTCACGAACCTTCTTCACCA |
| DN3407 | Aluminum activated malate transporter | F:TATACGACGGTTGCTTCACA  R:CCATCAACTCTGCCACAAAG |
| DN7768 | Actin | F:GTTGCTGGTGACTACGAGAA  R:ACCTCTCAAATCCAAGGCTG |

*Note: DN7768 (Actin) was selected as the reference gene, and the 2^⁻ΔΔCt^ method was used to calculate relative gene expression levels.*

Fig. S1 Effects of spore suspension concentrations on physiological indices of *C. chinensis* under 0.6% salt stress.

*(a) MDA content.*

*(b) SS content.*

*.*

Fig. S2 Maximum-likelihood phylogenetic tree of strain SZ01 based on concatenated *ITS* and *RPB2* gene sequences.

Numbers on branches indicate bootstrap support values calculated from 1000 standard bootstrap replicates. The scale bar represents a genetic distance of 0.02 substitutions per nucleotide site.

Fig S3 Colonial morphology of strain SZ01 cultured on PDA media supplemented with different concentrations of NaCl.

*(a).0%.*

*(b).5%.*

*(c).6%.*

*(d).7%.*

*(e).8%.*

*(f).9%.*

*(g).10%.*

Fig S4 qRT-PCR validation of selected DEGs from RNA-seq data.
